# Thromboses of major arteries and ChAdOx1 nCov-19 vaccination

**DOI:** 10.1186/s42466-021-00160-x

**Published:** 2021-11-29

**Authors:** Rujittika Mungmunpuntipantip, Viroj Wiwanitkit

**Affiliations:** 1 Bangkok, Thailand; 2 Dr DY Patil University, Pune, India

**Keywords:** Thromboses, Cov-19, Vaccination

## Abstract

This correspondence discussed on published article on “thromboses of major arteries and ChAdOx1 nCov-19 vaccination”.

Dear Editor, we would like to share ideas on the publication “Successful treatment of thromboses of major arteries after ChAdOx1 nCov-19 vaccination [1].” Zarrouk and Finsterer concluded that “*Both were successfully treated with intravenous immunoglobulins and non-heparin anticoagulant agents …. in case 1 and four months in case 2.* [1].” Thrombosis might occur in post COVID-19 vaccination. In the present case, the pathogenesis is still unclear. A possible mechanism in the present case is hyperviscosity following vaccination [2]. This is due to rapid change of blood viscosity due to immunogenicity process of COVID-19 vaccine [2]. The successful of treatment by non-heparin anticoagulant agents can support that the main pathological problem is not on coagulation component and platelet but plasma viscosity. Additionally, when there is a decline in stimulation, a decreased blood viscosity occurs and it is concordant with the observation that the patients improved within 6 weeks.

## Data Availability

Not applicable.

## Abbreviation

COVID-19: Coronavirus disease 2019
